# Evaluation of the Role of hsa-mir-124 in Predicting Clinical Outcome in Breast Invasive Carcinoma Based on Bioinformatics Analysis

**DOI:** 10.1155/2020/1839205

**Published:** 2020-03-03

**Authors:** Tongbao Feng, Ping Zhang, Yingxin Sun, Xiu Han, Jichun Tong, Zichun Hua

**Affiliations:** ^1^The State Key Laboratory of Pharmaceutical Biotechnology, College of Life Sciences, Nanjing University, Nanjing, China; ^2^Department of Clinical Laboratory, The Affiliated Changzhou No. 2 People's Hospital of Nanjing Medical University, Changzhou, China; ^3^Department of Cardiac Surgery, The Affiliated Changzhou No. 2 People's Hospital of Nanjing Medical University, Changzhou, China; ^4^Changzhou High-Tech Research Institute of Nanjing University and Jiangsu Target Pharma Laboratories Inc., Changzhou, China

## Abstract

**Purpose:**

Breast invasive carcinoma (BRCA) is the most common malignant tumor. MiR-124 plays a tumor-suppressive role in human cancer. However, the clinical significance of miR-124 in BRCA remains unclear. The aim of this study was to evaluate the association of hsa-mir-124 expression and the clinicopathological characteristics in BRCA using database analysis.

**Methods:**

The clinical data and expression profiles of hsa-mir-124 were obtained from the cancer genome atlas for BRCA (TCGA_BRCA). Then, the prognostic value of hsa-mir-124 in BRCA was investigated using the Cox Regression test, and the association of hsa-mir-124 and pathology TNM stages and pathologic stages were measured by the Kruskal–Wallis test and Wilcox. test. In addition, the association of hsa-mir-124 and tumor molecular phenotypes was performed using the Chi-Square test.

**Results:**

We found that the overall survival of patients with high expression of hsa-mir-124-1 and hsa-mir-124-2 was better than that of patients with low expression of hsa-mir-124-1 and hsa-mir-124-2. And the expression of hsa-mir-124-1, hsa-mir-124-2, and hsa-mir-124-3 was mainly enriched in T1/T2 stages, NO/N1 stages, and M0 stages. Then, the expression of hsa-mir-124-1, hsa-mir-124-2, and hsa-mir-124-3 was negatively associated with tumor lymph node metastasis. Moreover, the expression of hsa-mir-124 was associated with tumor molecular phenotype in breast invasive carcinoma.

**Conclusion:**

Our findings indicated that hsa-mir-124 expressions were associated with overall survival, TNM stages, pathologic characteristics, and tumor molecular phenotype in BRCA via TCGA_BRCA database, providing a new biomarker and a potential therapeutic target for BRCA patients.

## 1. Introduction

Breast tumor is one of the most common malignant tumors and the leading cause of cancer-related deaths among women worldwide [1, 2]. Using the classification scheme based on the World Health Organization (WHO) criteria, various histologic types of breast invasive carcinoma (BRCA) can be diagnosed by applying cytomorphologic criteria. Invasive ductal carcinoma (IDC) and invasive lobular carcinoma (ILC) account for about 90% of BRCA, of which IDC is the most common type, making up about 80% [3–5]. With IDC, cancer cells start in a milk duct, break through the walls, and invade breast tissue [4]. Previous studies reported that patients with BRCA had the worst prognosis of the breast cancer [6–8]. Therefore, it is urgently needed to identify new molecular markers to guide the diagnosis, treatment, and prognosis of BRCA.

Emerging studies have implicated that miR-124 plays the tumor-suppressive role in the development of the human cancer and acts as a diagnostic prognostic biomarker for cancer [9–13]. For instance, downregulation of miR-124 was an independent prognostic factor in patients with colorectal cancer [11, 14]. Moreover, miR-124-3p acted as a potential marker and suppresses tumor growth in gastric cancer [9]. In humans, three subtypes of hsa-mir-124 have been identified, namely, hsa-mir-124-1 (8p23.1), hsa-mir-124-2 (8q12.3), and hsa-mir-124-3 (20q13.33), with different chromosomal locations. However, the alterations in three subtypes of hsa-mir-124 profiles in different clinicopathological characteristics of BRCA are not yet fully understood.

In the present study, we investigated the expression of hsa-mir-124-1, hsa-mir-124-2, and hsa-mir-124-3 in breast invasive carcinoma by using bioinformatics analysis of the clinical parameters and survival data in TCGA_BRCA database. We found that the subtypes of hsa-mir-124 expressions were associated with overall survival and TNM stages in BRCA. In addition, we observed that the subtypes of hsa-mir-124 expressions were also associated with pathologic characteristics and tumor molecular phenotype in BRCA. These findings suggested that the alterations in hsa-mir-124-1, hsa-mir-124-2, and hsa-mir-124-3 profiles could provide a new biomarker and a potential therapeutic target for BRCA patients.

## 2. Materials and Methods

### 2.1. Data Acquisition

Linkedomics (http://www.linkedomics.org/) [15] was used to analyze the expression of hsa-mir-124 via a nonparametric test (attribute-dependent) in TCGA_BRCA. The expression of hsa-mir-124 was estimated by Human TCGA_BRCA miRNASeq HS miR 01/28/2016 Gene Firehose RPKM log2 and shown as (RPM, log 2 (Val + 1)) on the website. The clinical information (January 2016) regarding TCGA_BRCA was obtained from Human TCGA_BRCA Clinical 01/28/2016 Clinical Firehose on public databases. The clinicopathological data of 294 BRCA patients were used to analyze the association between the expression of hsa-mir-124-1 and hsa-mir-124-2 and the overall survival of BRCA patients; 742 BRCA patients were tested to analyze the association between the expression of hsa-mir-124-3 and the overall survival of BRCA patients. Then, the clinicopathological data of 754 patients, 744 patients, and 603 patients were enrolled to analyze the association of hsa-mir-124 expression and TNM stages, respectively, in BRCA patients. In addition, the clinicopathological data of 747 patients and 678 patients were enrolled to analyze the association of the expression of hsa-mir-124 and pathologic characteristics in BRCA. Moreover, the clinicopathological data of 43 patients and 44 patients were enrolled to analyze the association of the expression of hsa-mir-124 and tumor molecular phenotype in BRCA. And the demographic information and exclusion criteria for BRCA patients were according to the TCGA database.

### 2.2. Statistics Analysis

All the patients were divided into high and low expression groups according to the median expression of hsa-mir-124. The Cox regression test was used to investigate the prognostic value of hsa-mir-124 in BRCA. The Kruskal–Wallis test and Wilcox test were used to analyse the association of hsa-mir-124 and pathology TNM stages and pathologic stages in TCGA_BRCA. The chi-square test was performed to reveal the association of hsa-mir-124 and tumor molecular phenotypes in TCGA_BRCA. Differences with *p* < 0.05 were considered statistically significant.

## 3. Results

### 3.1. The Expression of hsa-mir-124 Was Associated with Overall Survival in Breast Invasive Carcinoma

**Figure 1:**
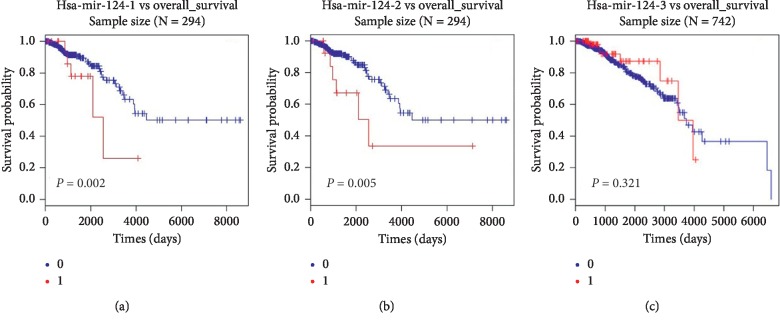
The expression of hsa-mir-124 was associated with overall survival in breast invasive carcinoma patients. The Cox regression test was performed to investigate the prognostic value of (a) hsa-mir-124-1, (b) hsa-mir-124-2, and (c) hsa-mir-124-3 in BRCA. Annotation: 0 (0 ≤ median, blue), 1 (1 > median, red).

To investigate the prognostic value of hsa-mir-124 in BRCA, we firstly performed the Cox regression test by deep sequencing data in TCGA_BRCA. The results showed that the overall survival of patients with high expression of hsa-mir-124-1 and hsa-mir-124-2 was better than that of patients with low expression of hsa-mir-124-1 and hsa-mir-124-2 (Figures 1(a) and 1(b), *N* = 294, *p* < 0.05). In addition, there was no statistically significant difference between the overall survival of patients with high expression of hsa-mir-124-3 and that of patients with low expression of hsa-mir-124-3 (Figure 1(c), *N* = 742, *p* > 0.05). These results suggested that the expression of hsa-mir-124 was associated with overall survival in breast invasive carcinoma patients.

### 3.2. The Expression of hsa-mir-124 Was Associated with TNM Stages in Breast Invasive Carcinoma

**Figure 2:**
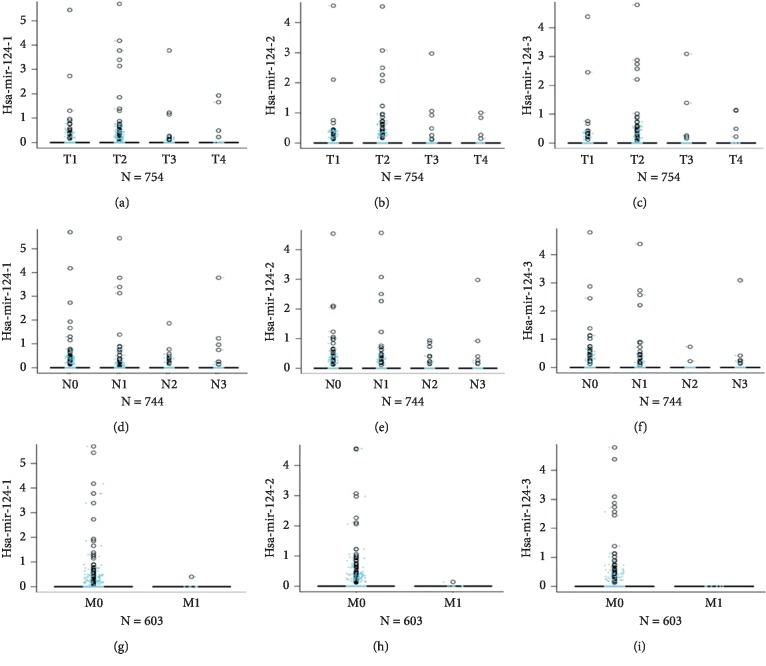
The expression of hsa-mir-124 was associated with TNM stages in breast invasive carcinoma. The Kruskal–Wallis test and Wilcox test were used to analyse the association of hsa-mir-124 and pathology TNM stages in TCGA-BRCA. (a) hsa-mir-124-1, (b) hsa-mir-124-2, and (c) hsa-mir-124-3 in T stage; (d) hsa-mir-124-1, (e) hsa-mir-124-2, and (f) hsa-mir-124-3 in N stage; and (g) hsa-mir-124-1, (h) hsa-mir-124-2, and (i) hsa-mir-124-3 in M stage.

To assess the pathological association between hsa-mir-124 and BRCA patients, we used the Kruskal–Wallis test and Wilcox test to analyse the association of hsa-mir-124 and pathology TNM stages in TCGA_BRCA. We found that the expression of hsa-mir-124-1, hsa-mir-124-2, and hsa-mir-124-3 in BRCA was mainly enriched in T1/T2 stages (Figures 2(a)–2(c), *N* = 754), N0/N1 stages (Figures 2(d)–2(f), *N* = 744), and M0 stages (Figures 2(g)–2(i), *N* = 603). These results indicated that hsa-mir-124-1, hsa-mir-124-2, and hsa-mir-124-3 primarily expressed in the early stages of BRCA development.

### 3.3. The Expression of hsa-mir-124 Was Associated with Pathologic Characteristics in Breast Invasive Carcinoma

**Figure 3:**
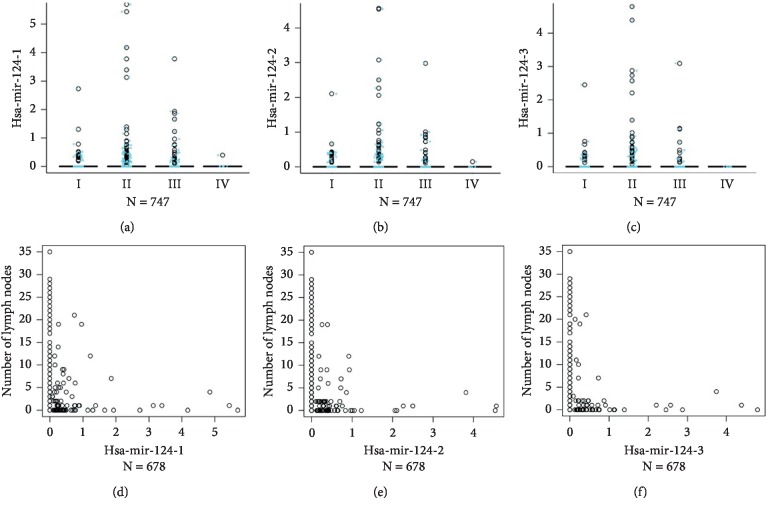
The expression of hsa-mir-124 was associated with pathologic characteristics in breast invasive carcinoma. The Kruskal–Wallis test was used to detect the association of hsa-mir-124 and pathologic stages in TCGA-BRCA. (a) hsa-mir-124-1, (b) hsa-mir-124-2, and (c) hsa-mir-124-3 in stage I/II/III/IV; (d) hsa-mir-124-1, (e) hsa-mir-124-2, and (f) hsa-mir-124-3 in lymph node metastasis.

To confirm the pathological association between hsa-mir-124 and BRCA patients, we performed the Kruskal–Wallis test to detect the association of hsa-mir-124 and pathologic stages in TCGA_BRCA. The data showed that the expression of hsa-mir-124-1, hsa-mir-124-2, and hsa-mir-124-3 in patients with BRCA was mostly concentrated in stage I/II (Figures 3(a)–3(c), *N* = 747). Moreover, we also performed spearman correlation to analysis the association of hsa-mir-124 and tumor lymph node metastasis in TCGA_BRCA. We found that the expression of hsa-mir-124-1, hsa-mir-124-2, and hsa-mir-124-3 was negatively associated with tumor lymph node metastasis in BRCA (Figures 3(d)–3(f), *N* = 678). These findings suggested that hsa-mir-124-1, hsa-mir-124-2, and hsa-mir-124-3 inhibited tumor growth in the BRCA development.

### 3.4. The Expression of hsa-mir-124 Was Associated with Tumor Molecular Phenotype in Breast Invasive Carcinoma

**Figure 4:**
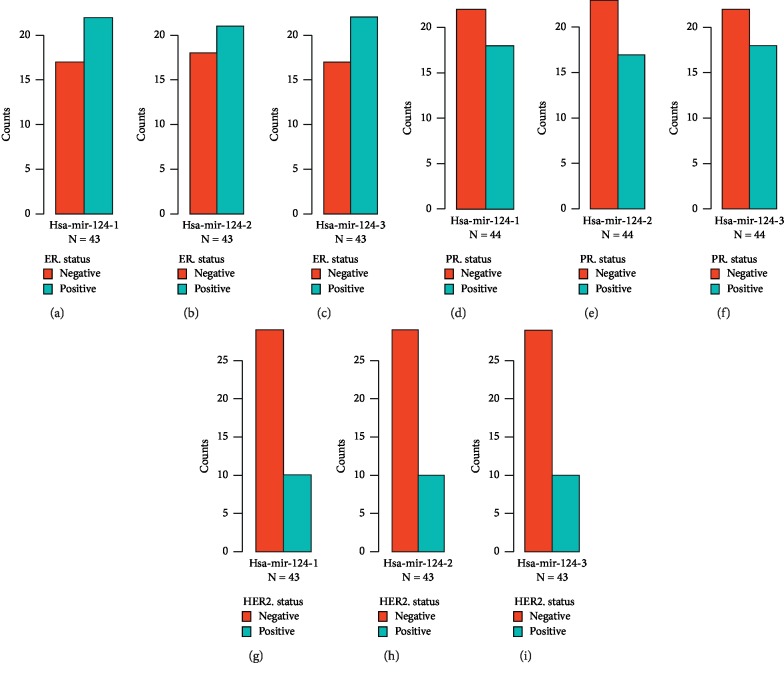
The expression of hsa-mir-124 was associated with tumor molecular phenotype in breast invasive carcinoma. (a) hsa-mir-124-1, (b) hsa-mir-124-2, and (c) hsa-mir-124-3 in ER status; (d) hsa-mir-124-1, (e) hsa-mir-124-2, and (f) hsa-mir-124-3 in PR status; and (g) hsa-mir-124-1, (h) hsa-mir-124-2, and (i) hsa-mir-124-3 in HER2 status.

To further evaluate the role of hsa-mir-124 in the progression of BRCA, we performed the chi-square test to reveal the association of hsa-mir-124 and tumor molecular phenotype in TCGA-BRCA. The results showed that the expression of hsa-mir-124-1, hsa-mir-124-2, and hsa-mir-124-3 in patients with estrogen receptor (ER) positive was higher than that in patients with ER negative (Figures 4(a)–4(c), *N* = 43). In addition, the expression of hsa-mir-124-1, hsa-mir-124-2, and hsa-mir-124-3 in patients with progesterone receptor (PR) negative was higher than that in patients with PR positive (Figures 4(d)–4(f), *N* = 44). Moreover, the expression of hsa-mir-124-1, hsa-mir-124-2, and hsa-mir-124-3 in patients with human epidermal growth factor receptor (HER2) negative was higher than that in patients with HER2 positive (Figures 4(g)–4(i), *N* = 43). These results indicated that hsa-mir-124 was associated with the molecular phenotype of BRCA tumors, which could be used to predict the molecular phenotype of tumors by detecting the expression of hsa-mir-124 in BRCA patients.

## 4. Discussion

As described in our research, we showed that noncoding RNAs were involved in breast cancer metastasis and miR-124 inhibited cell proliferation in breast cancer [16–18]. Consistent with our findings, miR-124 was decreased in colorectal tissue samples and was correlated with adverse clinical indicators and poor patient survival time [19–21]. Downregulation of plasma miR-124 expression was a predictive biomarker for prognosis of glioma [10, 22]. A large number of previous studies have suggested that miR-124 could be used as a molecular marker for tumor progression in cervical cancer [23, 24], breast cancer [12, 25, 26], lung cancer [27, 28], and osteosarcoma [29]. Numerous clinical studies have shown that BRCA is the most common type of breast cancer and has poor prognosis survival, and the pathogenesis of breast cancer involves a variety of factors and genes [4, 30]. Therefore, exploring the molecular diagnostic markers for BRCA could reduce cancer-related mortality and morbidity. In our present study, we investigated the significance of hsa-mir-124-1, hsa-mir-124-2, and hsa-mir-124-3 expression in breast invasive carcinoma by using the TCGA_BRCA database. We found that the subtypes of hsa-mir-124 expressions were associated with overall survival and TNM stages in BRCA. Moreover, we observed that the subtypes of hsa-mir-124 expressions were also associated with pathologic characteristics and tumor molecular phenotype in BRCA.

Based on the abovementioned findings, we concluded that the abnormal expression of hsa-mir-124 in BRCA was a favorable prognostic marker associated with less aggressive tumor characteristics. However, there were still some concerns that need to be expressed. Firstly, our conclusions were based on analysis and summary of the TCGA database, and we did not conduct further experimental analysis and verification. Secondly, our findings showed that hsa-mir-124 expressions were associated with overall survival, TNM stages, pathologic characteristics, and tumor molecular phenotype in BRCA, which were basically congruent with those of previous studies. However, this study was unable to determine the practical significance of hsa-mir-124 abnormal expression as a favorable prognosis marker and the relationship between existing known prognostic markers in BRCA. Thirdly, there was currently no effective targeted therapy for hsa-mir-124, which was questionable to evaluate the benefit of the aberrant expression of hsa-mir-124 for prognostic significance in BRCA. Therefore, in future studies, we need to further research the sensitivity and specificity of hsa-mir-124 in diagnosis of BRCA and explore the feasibility of targeted hsa-mir-124 in BRCA clinical treatment.

In summary, our present study indicated that hsa-mir-124 expressions were associated with overall survival, TNM stages, pathologic characteristics, and tumor molecular phenotype in BRCA via TCGA-BRCA database. These findings suggested that the alterations in hsa-mir-124-1, hsa-mir-124-2, and hsa-mir-124-3 profiles could predict clinical outcome in breast invasive carcinoma, which provided a molecular foundation to further research the development and progression of BRCA, and ultimately provided vital insights that may lead to personalized treatment of BRCA patients.

## Data Availability

The data used to support the findings of this study areincluded within the article.

## Abbreviations

BRCA: Breast invasive carcinoma.
IDC: Invasive ductal carcinoma.
ILC: Invasive lobular carcinoma.
TCGA: The Cancer Genome Atlas.
TNM: Stage, the primary tumor site and the regional lymph node involvement and the presence or otherwise of distant metastatic spread.
ER: Estrogen receptor.
PR: Progesterone receptor.
HER2: Human epidermal growth factor receptor.
